# Leaks can dramatically decrease FiO2 on home ventilators: a bench study

**DOI:** 10.1186/1756-0500-6-282

**Published:** 2013-07-21

**Authors:** Philippe Goutorbe, Erwan Daranda, Yves Asencio, Pierre Esnault, Bertrand Prunet, Julien Bordes, Bruno Palmier, Eric Meaudre

**Affiliations:** 1 Emergency, Anesthesia and ICU Departement, Military Teaching Hospital Sainte Anne, Boulevard Sainte Anne, Toulon 83000, France

**Keywords:** NIV, Leaks, Home ventilation, COPD, FiO2

## Abstract

**Background:**

Long term oxygen therapy improves survival in hypoxemic patients with chronic obstructive pulmonary disease (COPD). Because pressure support ventilation with a home care ventilator is largely unsupervised, there is considerable risk of leakage occurring, which could affect delivered FiO_2_. We have therefore conducted a bench study in order to measure the effect of different levels of O_2_ supply and degrees of leakage on delivered FiO_2_. Ventilator tested: Legendair® (Airox™, Pau, France). Thirty-six measures were performed in each four ventilators with zero, 5 and 10 l.min-1 leakage and 1,2,4 and 8 l O2 flow.

**Findings:**

FiO_2_ decreased significantly with 5 l.min-1 leakage for all O2 flow rates, and with 10 l.min-1 at 4 and 8 l.min-1 O2.

**Conclusion:**

During application of NIV on home ventilators, leakage can dramatically decrease inspired FiO2 making it less effective. It is important to know the FiO2 dispensed when NIV is used for COPD at home. We would encourage industry to develop methods for FiO2 regulation Chronic use of NIV for COPD with controlled FiO2 or SpO2 requires further studys.

## Background

Long-term oxygen therapy (LTOT) improves survival in hypoxemic patients with chronic obstructive pulmonary disease (COPD) [1,2]. The minimum recommended duration of O_2_ therapy is 15 hours per day. Non-invasive ventilation (NIV) is now recommended for acute on chronic COPD respiratory distress, whereas its chronic use is more controversial [3,4]. Nocturnal non-invasive ventilation (NIV) improves quality of life and blood gas status, with fewer intensive care admissions, although survival is not affected [5,6]. Most home care ventilators deliver pressure support via a turbine and a constant normobaric O_2_ supply [7,8]. The O_2_ source is liquid O_2_ or from extractors. We hypothesized that, whereas ICU ventilators deliver the set Fi02 (hyperbaric 02 witch permits variable O2 supply), with pressure support NIV, leakage around the mask could influence the received FiO2. Indeed leaks are well compensated by modern’s home ventilators by delivering higher flows, using an additional volume taken from the room air. Under constant O2 supply FiO2 should decrease. We therefore analysed the variation of delivered FiO_2_ in pressure support ventilation with a home care ventilator under different levels of O_2_ supply and leakage in a bench study.

## Material and method

### Experimental bench study

A Legendair® (Airox™, Pau, France) ventilator was set to give pressure support ventilation (PSV) with an expiratory pressure (PE) of 5 cmH2O and inspiratoty pressure (PI) of 15 cmH2O. A standard single circuit with valve was used to connect the ventilator with the test chamber. In place of the mask we used a ‘leaks valve’, which could allow 0, 5 or 10 l.min^-1^ leakage. The level of leakage was calibrated during a continuous airway pressure 10 cmH2O, with two pneumotachographs one before and one after the valve (Fleisch 6V, Lausanne, Switzerland). Signals were digitized by an analogic/digital system (MP150, Biopac Systems, Goleta, CA) with modules (DA 100V 1000z, Biopac System). The O2 supply was set on the ventilator and flow was measured by a FlowAnalyser PF-300 (Imtmedical, Switzerland).

**Figure 1:**
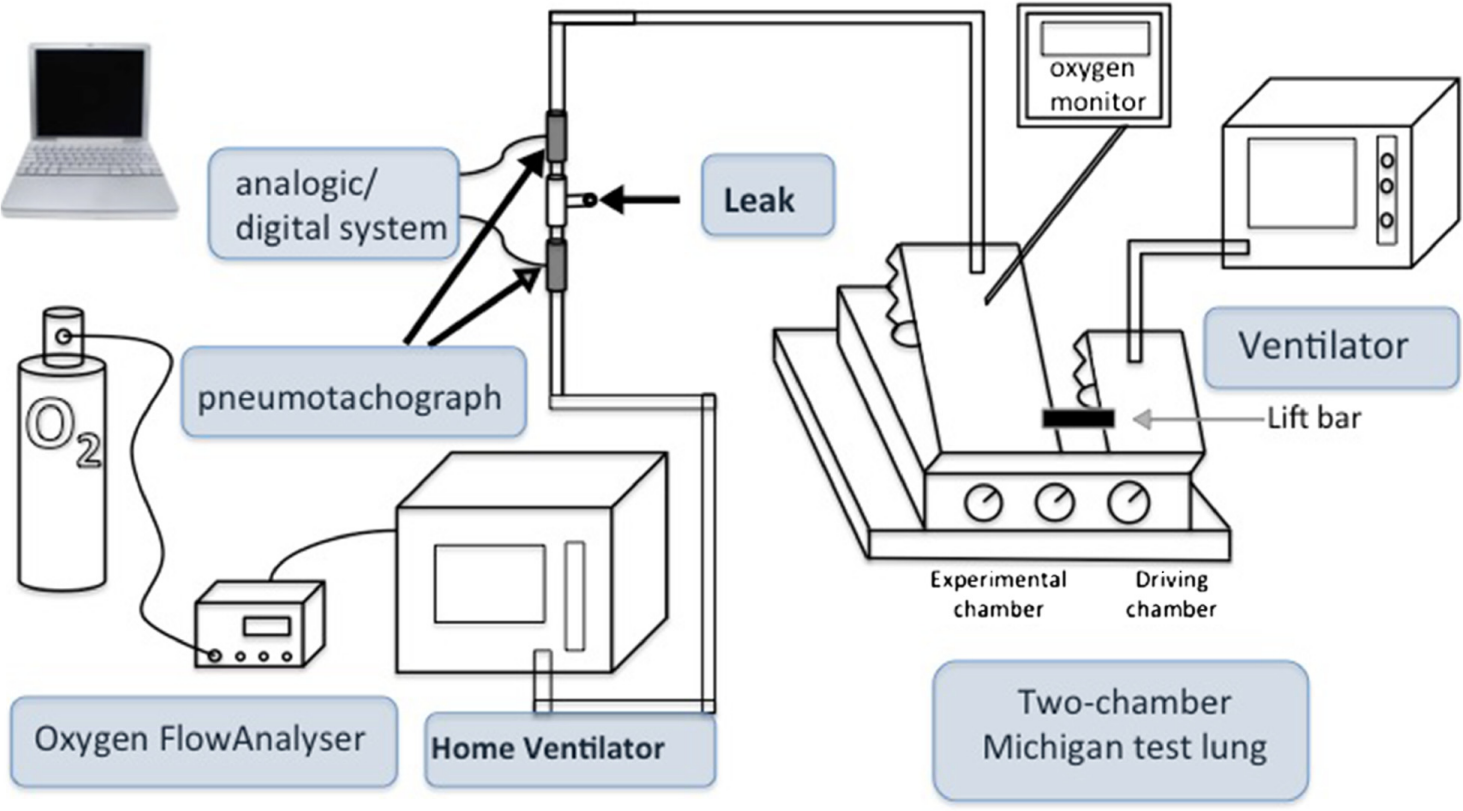
Show the bench test.

The test lung was the two-chamber Michigan test lung (Training/test lung-TTL®, adult infant lung simulator; Michigan Instrument™, Grand Rapids, MI), which has been described in detail in previous studies [9,10]; the infant lung was used as driving chamber and linked with the pressurized (test) chamber. The driving ventilator Puritan Bennet (PB) 840 (set in controlled ventilation) produces a negative pressure in the adult chamber, which is recognized as an inspiratory effort by the test ventilator. The characteristics of the test chamber were that of a parabolic airway resistor of 20-cmH2O l^-1^.^s-1^ (Pneuflo® Airway resistor; Michigan Instrument™, Grand Rapids, MI) and compliance was set at 60 ml/cmH2O. The respiratory rate of the driving ventilator was set to 15 per minute. The oxygen fraction in the test chamber was measured with a Puritan Bennet™ O2 Monitor 7820. We tested four flows of oxygen supply (1, 2, 4 and 8 liters/minute) and FiO_2_ for each with a 0, 5 and 10 liter/minute leak. The test chamber FiO_2_ was noted three times for each condition (Figure 1).

The Kruskal-Walis test was used to compare all variables and the *post hoc* Steel test if significance was found. All data are presented as group median [25–75]. Statistical analysis was performed with JMP 9.3 (SAS Institute, Cary, NC). Statistical significance was defined as *p* < 0.05.

## Findings

**Figure 2:**
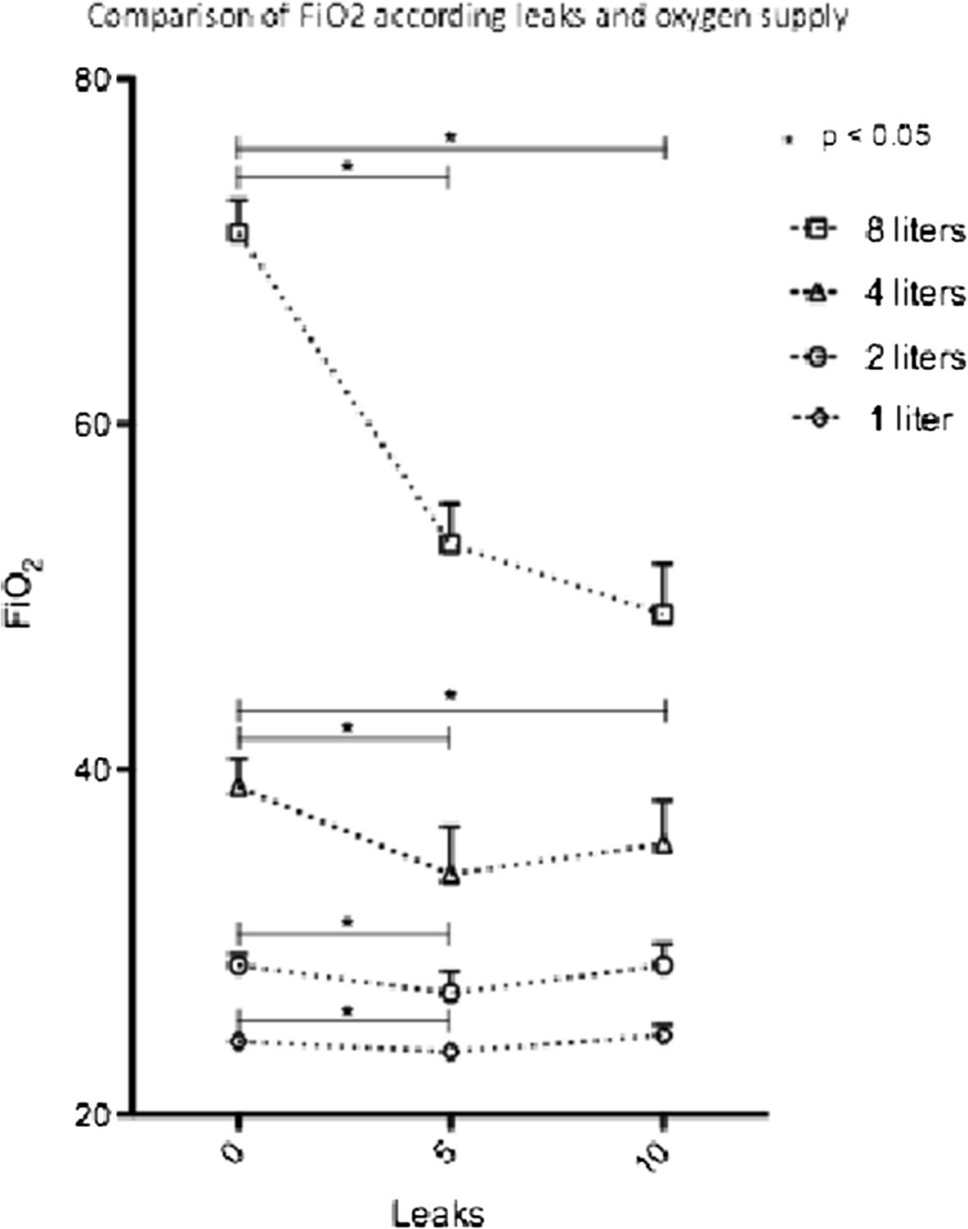
Show the variations of FiO2 with leaks.

Four different ventilators were tested three times each, with 12 measurements of FiO2 for each condition of oxygen supply and leakage. Leakage significantly affected FiO2 for all O2 supply between no leak and 5 liters per minute of leaks. FiO2 decreased significantly with 10 liters per minute of leaks only for 4 and 8 liters O2 supply (Table 1 and Figure 2).

**Table 1:** Comparison of FiO2 according leakages and oxygen supply

		**Leakage (liters per minute)**		
		**0**	**5**	**10**
		**n = 12**	**n = 12**	**n = 12**
	1 l/min	24.2 [23.7 - 24.4]	23.6 [23.2 - 23.9]*	25.1 [23.8 - 25.1]
Oxygen supply	2 l/min	28.6 [28.5 - 28.8]	27.0 [26.1 - 27.6]*	29.3 [27.4 - 29.6]
	4 l/min	39.0 [38.0 - 40.0]	34.0 [33.0 - 34.6]†	36.6 [34.5 - 38.0]*
	8 l/min	70.4 [67.5 - 71.9]	53.1 [51.2 - 54.0]†	49.5 [47.1 - 51.6]†

† p < 0.0001 ; * p < 0.05 compared to control group (no leakage).

## Discussion

The bench study confirms our hypothesis in six cases of eight; FiO2 decreases with all O2 flows with 5 liters per minute leaks and with 4 and 8 liters O2 supply with 10 liters per minute of leaks. We are not able to explain why in several conditions FiO2 increased with 10 liters per minute of leaks. We assume that it would be possible venturi effect with increased O2 flow but this one was continuously measured by a FlowAnalyser PF-300 (Imtmedical, Switzerland) and it was constant.

The Nocturnal Oxygen Therapy Trial (NOTT) and the Medical Research Council study, using similar inclusion criteria, demonstrated the beneficial effects of LTOT on survival in subjects with COPD and severe resting hypoxemia. The median survival in those using O2 for 15 hours/day was approximately twice that of those receiving no O2 [1,2]. The use NIV in acute exacerbation of COPD is now recommended. Two RCTs of long term NIV for respiratory failure failed to demonstrate any effect on mortality, although quality of life was improved and there were fewer hospital admissions. SPO_2_ at home was not reported [5,6].

Although some studies show no improvement in mortality [5,6,11], the physiological basis for NIV is clear; it relaxes the inspiratory muscles. External PEEP should counteract intrinsic PEEP [12], and VA/Q ratios may be improved [13]. However, leakage caused by movement of the mask is common. Opening the mouth, and poor fit to the face associated with weight loss, leads to leakage, especially at night [14]. Fortunately, many home ventilators now have well-developed software to detect and compensate for leaks from room air, and can compensate for leakage of about 30 l.min^-1^[15]. Nevertheless, leaks can decrease the delivered FiO_2_ because the O_2_ supply is constant, as demonstrated here. NIV failure to improve surviving in chronic COPD may be due to this.

There are no studies of the delivered FiO2 in NIV, the value of which is increased by oxygen supplementation with home ventilators. Thys and Schwartz showed the influence of site oxygen delivery on FiO2 [16,17]. Although in several home ventilators using a liquid O_2_ source or an O_2_ extractor, FiO_2_ can be monitored but it is not possible to set. Unfortunately regulation of FiO_2_ with home ventilators is not yet available. Our study is the first to focus on the affect of leakage on FiO_2_ during NIV with home ventilators, although such effects have been previously shown with ICU ventilators [18]. In several conditions FiO_2_ can vary by up to 30%. With the O2 supply of 8 l.min^-1^ we found a decrease in FiO_2_ from 70 to 50% with a leakage of 10 l.min^-1^.

NIV offers an excellent treatment for chronic respiratory insufficiency, but in the home there is no control of leaks or measurement of SPO_2_. In a recent meta-analyse Chen et al. point out that with an inspiratory positive airway pressure (IPAP) greater or equal than 14 cmH2O the PaO2 decreased [19]; increasing of leaks with high airway pressure should explain that. With increasing leakage, FiO_2_ decreases, and hypoxemia ensues leading to an increase of minute respiratory intake which in turn results in aspirating room air; if the O_2_ supply is constant then FiO_2_ decreases again leading a vicious circle of hypoxemia. This may explain the poor results of treating respiratory failure with NIV in the home.

The O_2_ supply can be triggered by the patient’s SpO_2_ or the ventilator FiO_2_. The former is more relevant clinically; the latter is more easily measured. The cost of measuring FiO_2_ is about 1000 Euros; SpO_2_ is less expensive but the measurement is subject to artefacts.

The design (bench test) limits the impact of the findings. On the over hand we underestimated the leaks, which could reach 30 liters per minute; the FiO2 decrease may be greater in clinical conditions.

We purpose to adjunct and O2 turbine witch should adjust O2 flow supply to a SpO2 objective.

New home NIV chronic use in COPD study’s should be conducted with this O2 flow control.

We propose to add a dedicated O2 turbine allowing automated O2 flow adjustment in order to achieve a predefined SpO2.

Thereafter, studies will be mandatory to evaluate effects of this automated O2 flow supply on COPD prognosis.

## Conclusion

This lung model study demonstrates that during application of NIV on home ventilators, leakage can dramatically decrease FiO2 making it less effective. As consequence, it is essential to know the FiO2 dispensed during NIV at home. Further research should explore the role of the O2 flow control during application of NIV in chronic hypercapnic COPD patients.

We hope that industry can address the problems we have highlighted in this study.

## Abbreviations

COPD: Chronicle obstructive pulmonary disease; FiO2: Inspired fraction of oxygen; IPAP: Inspiratory positive airway pressure; LTOT: Long term oxygen therapy; O2: Oxygen; NIV: Non invasive ventilation; NOTT: Nocturnal Oxygen Therapy Trial; PEEP: Positive end expiratory pressure; VA/Q: Ratio: ventilation perfusion ratio.

## Competing interest

The authors declare no competing interest.

## Authors’ contributions

GP drove the research and write the article, AY made the statistical analysis; and all the authors contributed to measurements. All authors read and approved the final manuscript.
